# Machine Learning-Driven D-Glucose Prediction Using a Novel Biosensor for Non-Invasive Diabetes Management

**DOI:** 10.3390/bios15030152

**Published:** 2025-03-01

**Authors:** Pardis Sadeghi, Shahriar Noroozizadeh, Rania Alshawabkeh, Nian Xiang Sun

**Affiliations:** 1 Electrical & Computer Engineering, W.M. Keck Laboratory for Integrated Ferroics, Northeastern University, Boston, MA 02115, USA; sadeghi.p@northeastern.edu (P.S.); alshawabkeh.r@northeastern.edu (R.A.); 2 Machine Learning Department, Carnegie Mellon University, Pittsburgh, PA 15213, USA; snoroozi@andrew.cmu.edu; 3 Heinz College, Carnegie Mellon University, Pittsburgh, PA 15213, USA; 4 Winchester Technologies, LLC, Burlington, MA 01803, USA

**Keywords:** diabetes, D-glucose, CNNs, RNNs, GANs, SMOTE, biosensors, molecularly imprinted polymer

## Abstract

Developing reliable noninvasive diagnostic and monitoring systems for diabetes remains a significant challenge, especially in the e-healthcare domain, due to computational inefficiencies and limited predictive accuracy in current approaches. The current study integrates machine learning with a molecularly imprinted polymer biosensor for detecting D-glucose in the exhaled breath condensate or aerosol. Advanced models, such as Convolutional Neural Networks and Recurrent Neural Networks, were used to analyze resistance signals, while classical algorithms served as benchmarks. To address challenges like data imbalance, limited samples, and inter-sensor variability, synthetic data generation methods like Synthetic Minority Oversampling Technique and Generative Adversarial Networks were employed. This framework aims to classify clinically relevant glucose levels accurately, enabling non-invasive diabetes monitoring.

## 1. Introduction

Diabetes is a widespread and critical health issue affecting millions globally, characterized by elevated blood sugar levels (hyperglycemia). The two primary types of diabetes have distinct causes: Type 1 diabetes occurs when the pancreas produces insufficient insulin due to damaged beta cells, while Type 2 diabetes results from the body’s inability to effectively use the insulin it produces. Both types can lead to severe complications if not managed properly [1]. Statistics indicate that the projected number of people living with diabetes worldwide is expected to reach more than 500 million by 2030. Additionally, around 84.1 million individuals have pre-diabetes, which, if untreated, can progress to Type 2 diabetes within five years. Also, it is estimated that 240 million individuals live with undiagnosed diabetes, with nearly half of all adults with diabetes being unaware of their illness [2,3]. Early detection is essential for preventing or delaying the progression of complications, especially in Type 2 diabetes. However, many individuals remain unaware of their condition until it has advanced. Despite the availability of various diagnostic methods, early detection of diabetes remains a challenge. The complexity of assessing multiple physiological parameters further complicates the diagnostic process, placing additional demands on healthcare professionals [4,5,6,7]. While conventional glucose monitoring techniques are effective, their invasiveness and associated discomfort often result in poor patient adherence. Frequent finger-prick testing is particularly burdensome, leading to irregular monitoring and delayed diagnosis. To address these limitations, we propose a non-invasive sensing platform that facilitates painless and user-friendly glucose monitoring. This approach is particularly advantageous for individuals at risk of developing diabetes or those managing Type 2 diabetes without insulin, as early identification of metabolic changes can enable timely lifestyle modifications and medical intervention, ultimately improving long-term disease management [1]. Table 1 presents a comparison of our proposed sensor with various MIP-based glucose sensors from the literature, focusing on the limit of detection.

Advanced techniques, such as data mining and machine learning, offer promising solutions to these limitations. By intelligently analyzing complex medical data, these technologies can aid in the early and accurate diagnosis of diabetes, improving outcomes and easing the decision-making process for physicians. Such advancements hold the potential to revolutionize the management and treatment of diabetes, emphasizing the need for innovative approaches in combating this global epidemic [13,14].

Recently, various algorithms have been applied to predict diabetes, encompassing traditional machine learning approaches [15] such as support vector machines (SVMs), decision trees (DTs), logistic regression, and others. Polat and Günes employed principal component analysis (PCA) combined with neuro-fuzzy inference to differentiate individuals with diabetes from those without [16]. Yue et al. utilized the quantum particle swarm optimization (QPSO) algorithm alongside a weighted least squares support vector machine (WLS-SVM) for type 2 diabetes prediction [17]. Additionally, Duygu and Esin developed a system called LDA-MWSVM for diabetes prediction [18]. In addition to traditional machine learning methods, recent studies have explored ensemble learning techniques and deep learning models for diabetes prediction. In [19], Jia et al. addressed the challenges of missing and imbalanced data in diabetes prediction by developing an ensemble method that integrates data imputation and balancing techniques such as SMOTE, leading to improved classification performance across multiple datasets. Moreover, Ganie et al. applied boosting algorithms to the Pima Indians diabetes dataset, demonstrating the effectiveness of ensemble methods in enhancing predictive performance [20]. Similarly, Fregoso-Aparicio et al. conducted a systematic review comparing 18 different models, finding that tree-based algorithms, including ensemble methods, demonstrated top performance in type 2 diabetes prediction [21]. Furthermore, deep learning approaches have shown promise; a systematic review highlighted their competitive performance in various aspects of diabetes prediction and management. Alam et al. conducted a comprehensive review of machine learning and artificial intelligence models in diabetes prediction and management, emphasizing the transformative potential of these technologies in enhancing predictive accuracy and patient care [22]. Additionally, in Ref. [23], Afsaneh et al. provided a comprehensive review of recent applications of machine learning and deep learning models in the prediction, diagnosis, and management of diabetes, underscoring the importance of these advanced techniques in improving healthcare outcomes.

Our research focuses on advancing sensors using functionalized molecularly imprinted polymers (MIPs) for detecting biomarker volatile organic compounds (VOCs) and small molecules in exhaled aerosols and condensate. These sensors are vital diagnostic tools for various diseases, including Alzheimer’s and lung cancer [24,25]. In early 2020, we redirected our efforts to develop MIP-based sensors for SARS-CoV-2 detection in a breathalyzer platform in response to the COVID-19 pandemic [26].

In this study [26], we developed and evaluated multiple detection schemes using machine learning and classical algorithms to classify experimental time-series data representing sensor responses. The dataset comprised 135 samples (63 positive and 72 negative), each labeled to indicate the presence or absence of SARS-CoV-2 pathogens in aerosols. Traditional methods, including wavelet decomposition and Gaussian mixture models, were chosen for their efficiency with limited training data, while a Long Short-Term Memory (LSTM) network represented the deep learning approach. Model performance was assessed through cross-validation and compared against human visual inspection. Additionally, we implemented a custom curve-fitting algorithm to identify significant sensor resistance changes across initialization, exposure, and recovery phases. This method relies on regression slopes and statistical measures to diagnose SARS-CoV-2 presence. Among the methods, the Long Short-Term Memory (LSTM) network achieved superior accuracy and balance between true positive and negative rates, though with increased computational demand. Conversely, wavelet-based classifiers offered a viable compromise for low-resource settings, and the curve-fitting approach achieved an impressive accuracy of 97.78%, underscoring the potential of integrating AI into sensor-based diagnostics [26].

**Figure 1:**
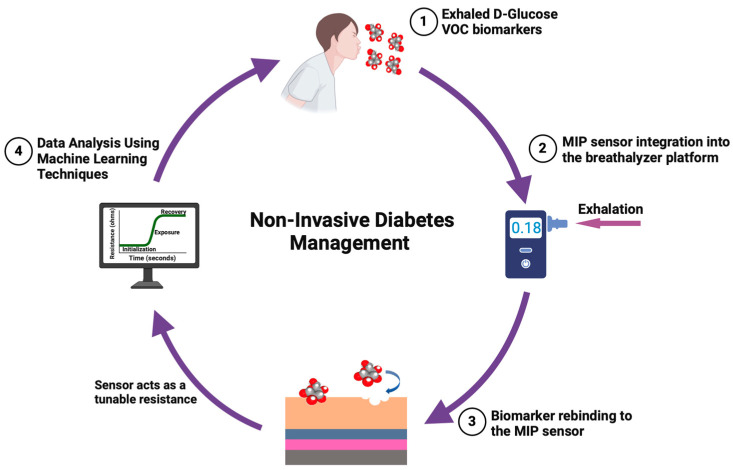
Schematic overview of study protocol and steps.

We have adapted this innovative technology for the selective detection of D-glucose aerosols in exhaled breath. The sensor functions by monitoring changes in ohmic resistance in response to aerosolized glucose, delivering highly accurate, specific, and sensitive results within 30 s. Featuring a low detection around 0.001 ppb, this sensor demonstrates exceptional potential for non-invasive diabetes management, enabling rapid and precise monitoring of glucose levels through breath analysis. Figure 1 illustrates the study protocol and the sequential steps involved in the procedure.

This current work additionally integrates a machine learning pipeline tailored to our biosensor data for glucose prediction. The emphasis will be on preprocessing techniques, which are essential in dealing with sensor noise, imbalances, and data inconsistencies. Advanced deep learning models like Convolutional Neural Networks (CNNs) and Recurrent Neural Networks (RNNs) will be applied to extract meaningful patterns in resistance signals, while classical machine learning approaches will be used as baseline comparisons. The final aim is to create a robust, accurate framework that can extend to different types of biosensor data to train a machine learning model that mimics a biosensor detecting multiple sugars in synthetic breath samples. Specifically, we need the model to classify glucose concentrations as either high or low, a distinction that is medically relevant for health monitoring. With data from different sensors, we also face inter-sensor variability, which can affect predictions if not accounted for. Limited sample sizes and the imbalance in high vs. low glucose classes further motivate our use of resampling strategies, including Synthetic Minority Oversampling Technique (SMOTE) and Generative Adversarial Networks (GANs), to synthesize additional training data. These resampling strategies are critical in creating a robust model that can generalize well to various biosensor readings.

Here, the focus is on making the raw biosensor data suitable for machine learning algorithms through thorough preprocessing steps. Baseline correction and normalization are critical due to the variabilities in sensor readings across time and different devices. Synthetic Minority Oversampling Technique (SMOTE) and Generative Adversarial Networks (GANs) will allow for resampling strategies, essential for dealing with imbalanced data in glucose readings. On the modeling front, classical machine learning models will serve as strong baselines, while deep learning models will exploit the time-series nature of the signals to achieve greater predictive power.

There are some anticipated challenges in this work, primarily around data variability and interpretability. Addressing these will ensure that the model is not only accurate but also robust across different environments and sensors. The impact of this research lies in creating a framework that not only advances glucose prediction but also sets the groundwork for biosensor-embedded machine learning, making it a highly adaptable and scalable solution for real-time health monitoring.

## 2. Materials and Methods

### 2.1. MIP Sensor Fabrication

Our electrochemical gas sensor integrates functionalized molecularly imprinted polymers (MIPs) with a graphene/Prussian-blue-coated substrate to achieve high specificity and sensitivity. All chemicals were obtained from Sigma Aldrich (St. Louis, MO, USA). Prussian blue (PB) enhances electrochemical sensitivity by facilitating efficient electron transfer with graphene (GR), creating a more porous and directional sensor structure. This improves the sensor’s limit of detection [27,28].

The functionalized molecularly imprinted polymer (MIP) layer is fabricated via electrochemical deposition using a potentiostat model, the Solartron SI 1287, creating imprinted cavities that precisely correspond to the target molecules in terms of size, shape, and functional group orientation. This configuration enables strong hydrogen bonding interactions, resulting in measurable changes in resistance upon target binding. The polymer matrix is electrochemically deposited around template molecules, and after the removal of the template, the imprinted cavities are tailored to the target molecule. Binding of the target reduces charge carrier mobility, leading to an increase in resistance, which is quantified using a digital multimeter. Sensor performance is assessed by monitoring resistance during the stabilization, exposure, and recovery stages.

The sensor fabrication begins with metal electrode deposition on a glass substrate was obtained from University Wafer (Boston, MA, USA), followed by the application of a graphene–Prussian blue layer to enhance conductivity. Polypyrrole (PPY) is electropolymerized in the presence of dopamine (DA) as the functional monomer, selected for its strong binding affinity and demonstrated specificity in detecting bovine hemoglobin [29]. DA undergoes self-polymerization into polydopamine (PDA), forming a stable, adhesive layer with broad substrate compatibility [20]. However, as PDA alone lacks conductivity, incorporating DA into PPY chains during electropolymerization ensures both adhesion and electrical conductance [30]. Hydrogen bonding between polydopamine and D-glucose enhances target recognition, leading to superior sensor performance compared to other functional monomers, such as methacrylic acid (MAA) [31]. Following electropolymerization, template molecules are removed via washing with 90% ethanol.

Scanning Electron Microscopy (SEM) analysis of MIP samples provided detailed insights into the sensor’s surface topography. To enhance image clarity, a 3 nm Gold–Palladium layer was sputter-coated onto the samples.

**Figure 2:**
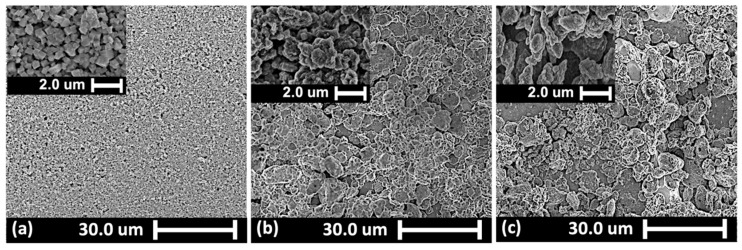
SEM images of MIP sensor surface (**a**) before electrodeposition, (**b**) after electrodeposition, and (**c**) after template removal.

Figure 2 presents SEM images of the sensor at different stages: before electrodeposition, after electrodeposition, and following template removal. Notable differences in surface topography are observed across these stages. The SEM image of the Gr-PB layer prior to electrodeposition (Figure 2a) reveals its initial morphology. After electrodeposition, a polymeric layer is visibly formed on the Gr-PB surface (Figure 2b). Additionally, the SEM image following template removal (Figure 2c) shows the presence of vacancies on the sensor’s surface, indicating the successful removal of the template.

### 2.2. Test Strategy

Our testing methodology relies on the binding of template molecule (D-Glucose) to the sensor, which immobilizes carriers in the semiconducting molecularly imprinted polymer layer, leading to reduced carrier mobility and a significant increase in ohmic resistance. To evaluate sensor performance, we use a newly developed 10 s–10 s–10 s testing protocol, as detailed in our previous publication [26]. This involves 10 s for sensor stabilization to establish a noise baseline, 10 s of exposure to aerosols containing varying virus loads or protein concentrations, and a final 10 s for recovery to observe resistance changes post-exposure. This streamlined approach delivers accurate measurements in just 30 s, offering a faster alternative to conventional methods without compromising reliability.

In the setup, the sensor (which rests on the middle of a PCB board) is faced down with an open Eppendorf vial containing the biomarker placed at 2–3 mm beneath the sensor. An ohmmeter monitors the sensor’s ohmic resistance during the operation. Our custom-designed test kit carefully designed to assess both the selectivity and performance of our glucose sensors. It encompasses 22 samples with both positive and negative concentrations. Among these, we have 16 D-Glucose kits, each covering a specific concentration range that serves as positive biomarkers with concentrations ranging from 90 ppm to 0.01 ppt. Additionally, the kit features 6 negative controls, consisting of common glucose analogs and potential interferents such as Arabinose and Fructose, with concentrations of 1 ppm, 10 ppm, and 15 ppm. In the context of molecularly imprinted polymer (MIP) sensors, selectivity testing focuses on molecules that closely resemble the size and structure of the template molecule, D-glucose, used in sensor fabrication. It is crucial to note that, given the aerosol nature of our context, these concentrations are in line with those found in saliva.

While our test kit comprises 22 distinct sample types (16 D-glucose concentrations and 6 negative sugar analogs), each sample type was tested multiple times, resulting in a total of 733 experimental time-series signals. Of these, among the D-glucose samples (584) approximately 25% had concentration of 80 ppm or higher (positive), and 75% had concentration lower than 80 ppm, yielding a positive-to-negative class ratio of about 1:3 for D-glucose. This experimental dataset forms the basis for our machine learning models.

### 2.3. Data Preprocessing

We rely on an experimental dataset of time series captured for different glucose concentrations that were acquired as noted in the prior section. Each dataset represents a 30 s observation sampled at a frequency of approximately 1.92 Hz. To ensure that the data were suitable for both deep learning models (CNNs and RNNs) and classical machine learning models, we employed a multi-step preprocessing pipeline consisting of interpolation, smoothing, baseline correction, normalization, and resampling.

#### 2.3.1. Time Window Selection

To focus the analysis on the most relevant part of the signal, we selected a specific time window from 8 to 22 s. This time window was chosen based on sensor dynamics, which indicated that the majority of the signal stabilization and glucose response occurred within this period. Data outside this window were excluded.

#### 2.3.2. Baseline Correction

Each signal had an inherent baseline variation due to sensor-specific noise. To account for this, we performed baseline correction by subtracting the baseline value from the entire signal. The baseline was computed as the average resistance value within the first 6 s of the signal, where glucose concentration was assumed to be stable:

(1) $$Baseline=\frac{1}{T_{b}}\sum_{t=0}^{T_{b}}R\left(t\right)$$

where $T_{b}$ is the time window used for baseline calculation and *R*(*t*) is the resistance value at time *t*. This baseline correction step helped in normalizing the initial sensor readings across different sensors, ensuring that the subsequent analysis focused on the relative changes in resistance.

#### 2.3.3. Smoothing and Interpolation

The raw sensor signals often contained noise and missing data points. To handle this, we applied a smoothing filter to reduce high-frequency noise, followed by linear interpolation to ensure consistent signal lengths across all samples. Specifically, the following interpolation method was used:

(2) $$R^{\prime }\left(t\right)=R\left(t_{1}\right)+\frac{\left(R\left(t_{2}\right)-R\left(t_{1}\right)\right)\cdot \left(t-t_{1}\right)}{t_{2}-t_{1}}$$

where *t*_1_ and *t*_2_ are consecutive time points with known resistance values, and *R*′(*t*) is the interpolated resistance at time *t.* The smoothed and interpolated signals were then resampled to ensure that all signals had the same number of timesteps, facilitating batch processing in deep learning models.

#### 2.3.4. Normalization

To ensure that the machine learning models could effectively learn from the data, the resistance values were normalized to have zero mean and unit variance:

(3) $$R_{\mathrm{norm}}\left(t\right)=\frac{R\left(t\right)-\mu }{\sigma }$$

where *µ* and σ represent the mean and standard deviation of the resistance values, respectively. Normalization ensures that all features are on the same scale, which helps improve the convergence behavior of gradient-based optimization algorithms used in training deep learning models.

### 2.4. Resampling Techniques for Class Imbalance

Given the imbalance between the high glucose (≥80 ppm) and low glucose (<80 ppm) classes, we employed several resampling techniques to ensure that the models could learn from both classes equally:

- Oversampling of Minority Class: This method involves duplicating existing samples from the minority class to match the number of samples in the majority class. Although simpler than the resampling methods that follow, oversampling can still provide a balanced dataset, allowing the models to focus equally on both classes.
- SMOTE (Synthetic Minority Oversampling Technique): SMOTE was used to synthesize new samples for the minority class by interpolating between existing samples:

(4) $$x_{\mathrm{new}}=x_{\mathrm{minority}}+\lambda \cdot \left(x_{\mathrm{neighbor}}-x_{\mathrm{minority}}\right)$$

where $x_{\mathrm{minority}}$ is a sample from the minority class, $x_{\mathrm{neighbor}}$ is one of its k-nearest neighbors, and *λ* is a random number between 0 and 1. This method helped in artificially expanding the minority class without duplicating samples.

- Generative Adversarial Networks (GANs [32]): A GAN was trained to generate synthetic data that mimicked the real sensor data. The generator creates synthetic data samples, while the discriminator attempts to distinguish between real and synthetic data. The generator and discriminator loss functions used are defined as follows:

(5) $$L_{D}=-E_{x∼p_{data}}\left[\log D\left(x\right)\right]-E_{z∼p_{z}}\left[\log\left(1-D\left(G\left(z\right)\right)\right)\right]$$

(6) $$L_{G}=-E_{z∼p_{z}}\left[\log D\left(G\left(z\right)\right)\right]$$

where *D*(*x*) is the probability that *x* is real and *G*(*z*) is the synthetic data generated from noise *z*. After training, synthetic samples from the GAN were added to the minority class, balancing the dataset.

### 2.5. Feature Extraction for Classical Models

For classical machine learning models such as Random Forest, XGBoost, and SVM, we extracted several statistical features from the time-series data. These features summarize the signal behavior over time, allowing classical models to work on structured data. The following features were extracted:

- Mean and Standard Deviation: These basic statistical features provide information about the average resistance value and its variability:

(7) $$\mathrm{Mean}=\frac{1}{N}\sum_{i=1}^{N}R\left(t_{i}\right)$$

(8) $$\mathrm{Standard}\;\mathrm{Deviation}=\sqrt{\frac{1}{N}\sum_{i=1}^{N}{\left(R\left(t_{i}\right)-\mu \right)}^{2}}$$

- Min and Max Values: The minimum and maximum values of the signal are critical for identifying extreme fluctuations in resistance, which may correspond to glucose peaks or drops.
- Energy: The energy of the signal is computed as the sum of squared resistance values over time, providing an indication of the overall signal strength:

(9) $$\mathrm{Energy}=\sum_{i=1}^{N}R{\left(t_{i}\right)}^{2}$$

- Skewness and Kurtosis: These features capture the shape of the distribution of resistance values. Skewness measures the asymmetry of the distribution, while kurtosis measures the “tailedness”:

(10) $$\mathrm{Skewness}=\frac{\frac{1}{N}\sum_{i=1}^{N}{\left(R\left(t_{i}\right)-\mu \right)}^{3}}{\sigma^{3}}$$

(11) $$\mathrm{Kurtosis}=\frac{\frac{1}{N}\sum_{i=1}^{N}{\left(R\left(t_{i}\right)-\mu \right)}^{4}}{\sigma^{4}}-3$$

- Fourier Transform Features: To capture frequency domain characteristics, we applied the Fast Fourier Transform (FFT) and extracted the mean and standard deviation of the FFT magnitude spectrum:

(12) $$R\left(f\right)=\sum_{t=1}^{T}R\left(t\right)e^{-j2\pi ft}$$

where *R*(*f*) represents the Fourier-transformed signal at frequency *f* and *T* is the length of the time window. These spectral features provide insights into the periodic behavior of the signal, which may be indicative of sensor noise or glucose dynamics.

### 2.6. Modeling Approaches

In this study, we employed a combination of deep learning and classical machine learning approaches to predict glucose concentrations from biosensor data. Our deep learning models included Convolutional Neural Networks (CNNs) and Recurrent Neural Networks (RNNs), both of which are well-suited for analyzing time-series data like the resistance signals generated by the biosensor. These models were chosen due to their ability to capture temporal and spatial patterns in sequential data. For the deep learning models, we transformed the preprocessed time-series signals into formats suitable for the CNN and RNN architectures. The CNN was used to capture local dependencies in the data by applying filters across the time axis, while the RNN, specifically the Long Short-Term Memory (LSTM) network, was used to retain long-term temporal dependencies in the signals. Both models were trained with cross-entropy loss for binary and multiclass classification tasks.

Additionally, we explored classical machine learning models such as Random Forest, XGBoost, and Support Vector Machines (SVM), where we extracted handcrafted features from the preprocessed signals and applied these models to the resulting structured data. These classical models were selected for their robustness and ability to handle structured data effectively. In the following subsections, we describe in detail the architecture of the CNN and RNN models, the preprocessing steps that enabled their application to time-series data, and the rationale behind the classical models used for comparison.

Each time series is recorded over a 30 s interval, but only the 8–22 s window is used (≈14 s total). With a sampling rate of approximately 1.92 Hz, each signal contains around 27 data points after preprocessing. For the CNN, we format the input as (*batch_size*, 1, 27) to allow for 1D convolution over the time dimension. For the LSTM, the input is reshaped to (*batch_size*, 27, 1) to represent sequences of 27 time steps.

#### 2.6.1. Convolutional Neural Networks (CNNs)

CNNs are particularly effective in processing time-series data because they can capture local patterns and hierarchies. We used a 1D CNN architecture, with each convolutional layer followed by a pooling layer to reduce the dimensionality. The architecture is as follows:

- Input Layer: The input to the CNN is the normalized time-series data, shaped as (N, 1, T), where N is the batch size and T is the number of timesteps (after interpolation).
- Convolutional Layers: The first convolutional layer applies a 1D convolution with a kernel size of 5 and 16 filters. This is followed by another convolution with 32 filters:

(13) $$h=\mathrm{ReLU}\left(W\cdot x+b\right)$$

where *h* is the output of the convolution, *W* is the kernel, *x* is the input, and *b* is the bias term.

- Max Pooling: A pooling layer is applied to downsample the feature maps:

(14) $$p\left(t\right)=\max\{h\left(t\right),h\left(t+1\right),\ldots ,h\left(t+k-1\right)\}$$

where *k* is the pooling window size.

- Fully Connected Layers: After flattening the feature maps, fully connected layers are used to map the extracted features to the output (binary or multiclass).
- Dropout: A dropout layer with 50% probability is added to prevent overfitting.

A dropout rate of 50% was applied in the final fully connected layers to prevent overfitting, in line with the original recommendations of [33,34,35], where dropout rates between 20% and 50% are commonly cited for tasks with limited training data. Empirically, we tried dropout rates ranging from 10–50% and found 50% dropout minimized validation loss in our experiments, which aligns with findings in smaller-scale datasets.

#### 2.6.2. Recurrent Neural Networks (RNNs)

RNNs, particularly Long Short-Term Memory (LSTM) networks, are ideal for capturing temporal dependencies in sequential data. Our LSTM model consists of two layers with 32 hidden units each. The LSTM is designed to retain long-term dependencies in the glucose data, which is crucial for predicting trends in glucose concentrations. The hidden state $h_{t}\;$ and cell state $c_{t}$ are updated at each time step as follows:

(15) $$f_{t}=\sigma \left(W_{f}\cdot \left[h_{t-1},x_{t}\right]+b_{f}\right)\;i_{t}=\sigma \left(W_{i}\cdot \left[h_{t-1},x_{t}\right]+b_{i}\right)\;o_{t}=\sigma \left(W_{o}\cdot \left[h_{t-1},x_{t}\right]+b_{o}\right)\;c_{t}=f_{t}*c_{t-1}+i_{t}*\tanh\left(W_{c}\cdot \left[h_{t-1},x_{t}\right]+b_{c}\right)\;h_{t}=o_{t}*\tanh\left(c_{t}\right)$$

where $f_{t}$, $i_{t}$, and $o_{t}$ are the forget, input, and output gates, respectively, and *c_t_* is the cell state. These gates control the flow of information through the LSTM, allowing it to retain important temporal information across timesteps.

#### 2.6.3. Classical Machine Learning Models

- Random Forest: A Random Forest model was trained on the extracted features. The ensemble of decision trees helps to improve model robustness and reduce overfitting. Each decision tree is trained on a random subset of the data, and the final prediction is the majority vote of all trees.
- XGBoost: XGBoost is a gradient boosting algorithm that builds trees sequentially. Each new tree is trained to correct the errors made by the previous trees. XGBoost incorporates regularization to control overfitting and is well-suited for imbalanced datasets.
- SVM: We employed SVMs with both linear and RBF kernels. SVMs find the hyperplane that best separates the classes in the feature space, maximizing the margin between the classes. For imbalanced datasets, SVMs are sensitive to the regularization parameter C, which controls the tradeoff between maximizing the margin and minimizing classification errors.

## 3. Results

### 3.1. Biosensor

**Figure 3:**
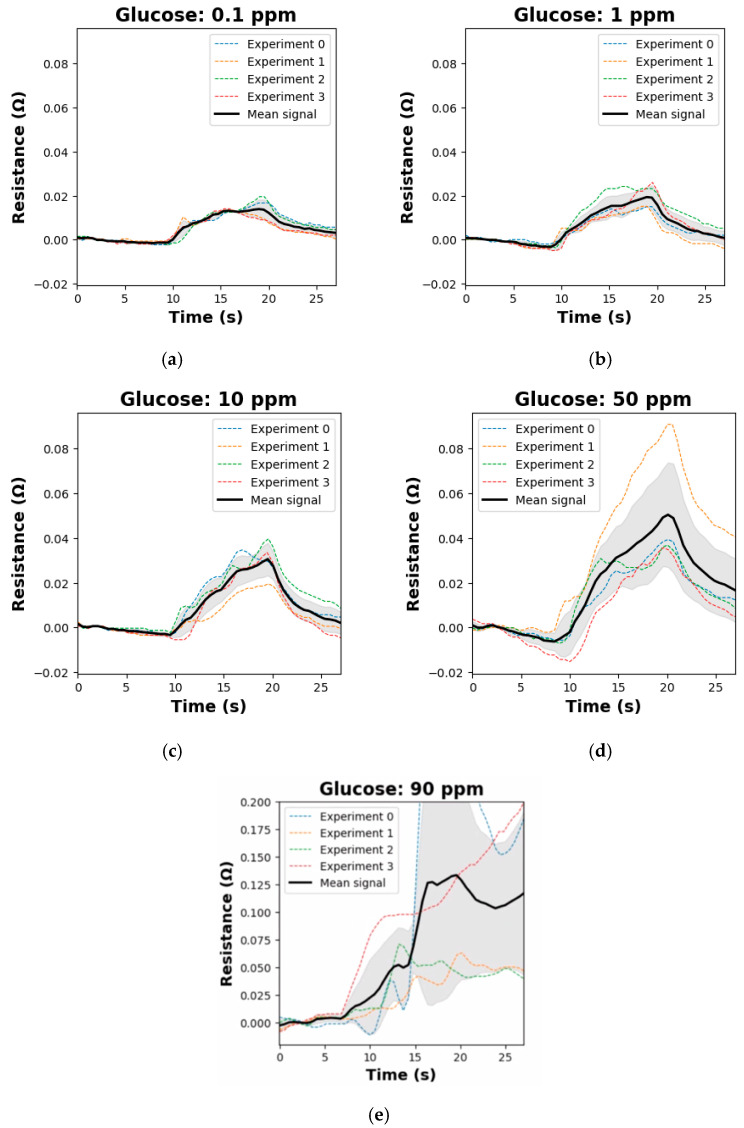
Sensor resistance change as a function of D-glucose concentration. Panels represent data for concentrations of (**a**) 0.1 ppm, (**b**) 1 ppm, (**c**) 10 ppm, (**d**) 50 ppm, and (**e**) 90 ppm.

Glucose concentrations in exhaled breath condensate (EBC) and aerosols vary widely across the literature. A key distinction of this work, compared to our other study currently under review (P. Sadeghi et al., “Real-Time Detection of D-Glucose Molecules in Exhaled Aerosols Using a Biochemical Sensor for Breathalyzer Applications”, submitted to IEEE Sensors Letters) [36], lies in its broader focus. While the other study presents a narrower scope, concentrating on breath aerosols and EBC, achieving a Pearson correlation value of 0.92 and incorporating human trials, this study extends to three specific mediums: EBC, aerosols, and saliva. Using advanced test kits, this work places greater emphasis on machine learning methods to enhance analysis. Our sensor demonstrates a unique capability to detect D-glucose in saliva, further highlighting its potential for targeted disease monitoring. To address reported variability, we tested the sensor across a concentration range from 90 ppm to 0.001 ppb, encompassing the highest and lowest values documented in the literature. The detection limit (S/N = 3) was determined at lower concentrations, and each sensor was evaluated for D-glucose sensitivity. The sensors limit of detection is approximately below 0.001 ppb. Figure 3 presents examples of the relationship between sensor resistance change and D-glucose concentration, based on data from 733 experiments. The normalized results illustrate the mean resistance change observed at each concentration.

**Figure 4:**
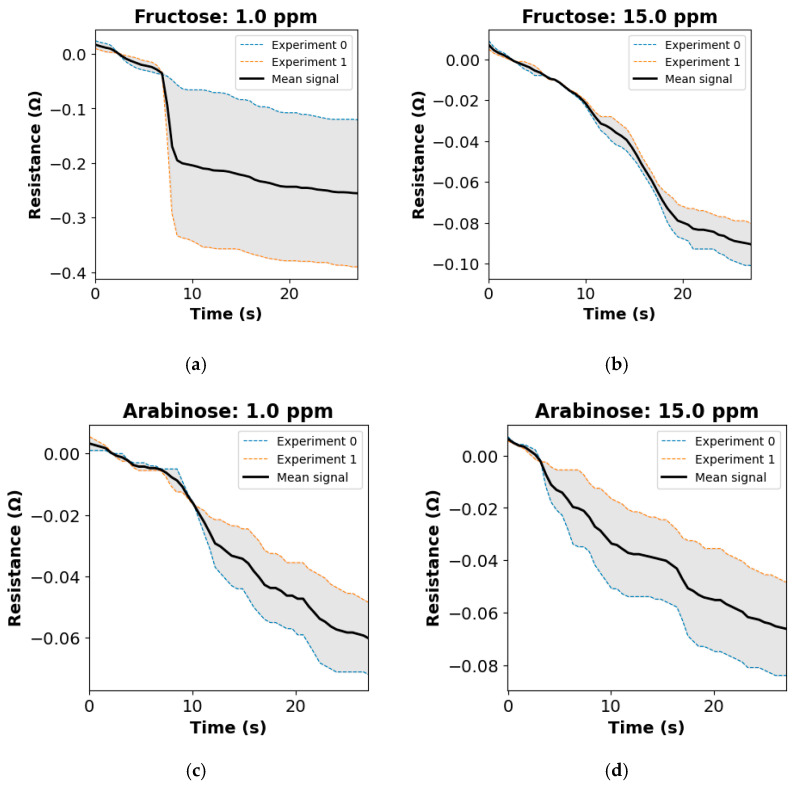
Sensor resistance change as a function of negative control concentrations. Panels represent data for concentrations of (**a**) Fructose 1 ppm, (**b**) Fructose 15 ppm, (**c**) Arabinose 1 ppm, and (**d**) Arabinose 15 ppm.

Figure 4 showcases some selectivity test results, demonstrating that glucose analogs caused negative shifts in sensor resistance. While further testing is needed to evaluate potential interference from other volatile organic compounds (VOCs) in exhaled breath, trials with healthy participants across various physiological states showed consistent and clear glucose detection. These findings suggest minimal interference from other VOCs, confirming the strong selectivity of our sensor for D-glucose.

To assess the reusability, sensors were rinsed with 90% ethanol for 3 min, dried, and retested over the course of a week. As an example, a sensor with an initial resistance of 35.62 Ω was tested at 1 ppm glucose, showing average resistance changes of 0.0213 Ω in week 1, 0.0224 Ω in week 2, and 0.0232 Ω in week 3, all within the expected range. By week 4, the change increased to 0.25 Ω, reaching 0.027Ω by the end of the month, indicating a decline in accuracy. Testing during the first three weeks ensures optimal sensor performance.

### 3.2. Machine Learning Prediction Task

- Binarization of Glucose Concentrations and Other Sugar Types

The prediction task in this study was designed to classify glucose concentrations and differentiate them from other substances measured by the biosensor. Specifically, glucose concentrations were binarized into two clinically relevant categories: low glucose (less than 80 ppm) and high glucose (80 ppm or greater). This binarization reflects the physiological importance of glucose thresholds in managing conditions such as hypoglycemia and hyperglycemia.

Research indicates that postprandial salivary glucose levels are elevated in individuals with diabetes. In a study that examined both controlled and uncontrolled diabetics, the mean postprandial salivary glucose (PPSG) levels for uncontrolled diabetic patients were approximately 80 ppm, while controlled diabetics had a mean PPSG of approximately 50 ppm. This threshold helps identify hypoglycemia, requiring immediate intervention, while hyperglycemia warrants monitoring rather than urgent action [37]. The 80 ppm threshold was selected based on clinical guidelines, which indicate that glucose concentrations below this threshold may require immediate medical intervention to prevent hypoglycemic events, whereas concentrations above this threshold are less critical in immediate decision-making but may still require monitoring for potential hyperglycemia. By discretizing glucose into these two classes, we align the prediction task with clinically actionable thresholds, facilitating the development of predictive models that can be used in real-time biosensor applications. Additionally, in the multiclass setup, we included other substances such as fructose and sucrose without binarizing their concentrations. This allows us to investigate the sensor’s ability to distinguish glucose from other sugars, a crucial aspect for practical applications where distinguishing between glucose and other sugar types is essential.

- Rationale for Prediction Task Setup

The choice of binarizing glucose concentrations while keeping other substances intact in a multiclass setup was driven by a combination of clinical and biological motivations. First, glucose management is the primary target of biosensor applications, as glucose concentration directly correlates with diabetic monitoring. However, the ability to detect and distinguish glucose from other substances is equally important in ensuring the sensor’s specificity and sensitivity. In real-world applications, biosensors must accurately differentiate glucose from similar sugars, such as fructose, which can be present in biological samples. Our experimental design is thus tailored to both the clinical focus on glucose and the broader biochemical context in which the sensor operates.

### 3.3. Evaluation Criteria and Metrics

To evaluate the performance of the machine learning models developed in this study, we used a combination of standard classification metrics that account for the imbalanced nature of the dataset and the multiclass prediction setup. These metrics were selected to provide a holistic view of the model’s performance in terms of both overall accuracy and the handling of individual classes (especially the minority class in binary glucose classification).

- Accuracy: The proportion of correctly classified instances.
- Precision: The proportion of true positive predictions among all positive predictions.
- Recall: The proportion of actual positive instances correctly identified.
- F1-Score: The harmonic mean of precision and recall.
- Confusion Matrix: A matrix that summarizes the classification results.

In our machine learning analysis, we evaluated various models for glucose concentration classification using different preprocessing techniques to address issues such as data imbalance and inter-sensor variability. The performance of each model was assessed based on accuracy, F1-score, precision, and recall that can be seen in Table 2.

For the deep learning models, the Convolutional Neural Network (CNN) and Long Short-Term Memory (LSTM) models, performance varied across preprocessing methods. For both CNN and LSTM, the baseline model without any resampling achieved comparable results with an accuracy of 0.6778, indicating moderate predictive capability even without resampling techniques. However, the application of Minority Oversampling improved the F1-score in CNN (0.5211) and yielded a slight enhancement in recall in both CNN and LSTM models, highlighting the value of oversampling for better representation of the minority class. Conversely, applying SMOTE or GAN-based resampling generally led to a decline in accuracy for the deep learning models, suggesting that these methods may introduce noise rather than useful synthetic data in these contexts. For classical models, the Random Forest classifier generally outperformed other classical models in accuracy, reaching 0.7197 with SMOTE preprocessing, accompanied by a relatively higher F1-score of 0.4643 and notable recall improvement. XGBoost and SVM models showed moderate improvements with SMOTE and Minority Oversampling but achieved lower accuracy and F1-scores compared to Random Forest. Notably, XGBoost showed its highest F1-score with SMOTE (0.4083), whereas SVM’s performance was more inconsistent, reaching its best F1-score of 0.4327 with Minority Oversampling, despite a lower overall accuracy. The results indicate that Random Forest with SMOTE preprocessing was the most effective classical machine learning configuration in terms of both accuracy and F1-score. Meanwhile, the deep learning models, specifically CNN and LSTM, achieved comparable accuracy without extensive preprocessing, highlighting their potential suitability in glucose concentration prediction tasks where feature extraction is limited.

## 4. Discussion

The findings from the machine learning portion of this study demonstrate that preprocessing strategies and model selection play significant roles in the predictive performance of machine learning models for glucose concentration classification using biosensor data.

The deep learning models (CNN and LSTM) achieved similar performance metrics across different preprocessing methods, indicating a degree of robustness to data imbalance. These models achieved moderate accuracy without resampling, suggesting that deep learning architectures can directly process raw biosensor signals without extensive data augmentation or feature engineering. However, the application of GAN-based resampling did not improve performance, likely due to challenges in generating realistic synthetic samples for high-dimensional, sensor-derived data. Future work could explore improved GAN architectures and diffusion models, specifically designed to handle time-series data, to better capture the intricate patterns in glucose concentration signals.

Among the classical machine learning models, Random Forest with SMOTE preprocessing emerged as the top performer, achieving the highest accuracy and recall. The structured features extracted from the time-series signals provided the Random Forest classifier with meaningful input, allowing it to outperform other classical methods. SMOTE was especially effective in balancing the class distribution, enhancing the recall in Random Forest and XGBoost models. XGBoost also benefited from SMOTE, albeit with slightly lower performance than Random Forest, highlighting its sensitivity to class imbalance. Interestingly, SVM exhibited variable performance with different preprocessing methods, indicating that it may be less suited for this specific biosensor data compared to other classifiers. The poor performance of SVM with GAN-based resampling further suggests that the synthetic data generated may not accurately reflect the characteristics of the original glucose data.

Despite these promising results, one notable limitation is the relatively low F1-scores across models, which reflect the challenges posed by our limited dataset size and the class imbalance inherent in our glucose concentration data. Although we applied resampling techniques like SMOTE and GANs to mitigate the imbalance, the limited number of high-concentration glucose samples restricted the models’ capacity to generalize to unseen data, particularly in high glucose categories. This imbalance resulted in models that, while somewhat accurate in overall classification, struggled to capture the minority class effectively, which impacted the F1-scores. Addressing this issue fully would likely require a larger, more representative dataset, particularly to enhance the model’s ability to identify glucose at clinically relevant thresholds.

While data augmentation strategies like SMOTE and GAN offered incremental improvements, their effectiveness was constrained by the limited number of original samples from which synthetic data could be generated. Specifically, generating realistic and diverse synthetic samples for high-dimensional biosensor data proved challenging with the current approaches, potentially introducing artifacts rather than useful variability. Future research could focus on developing more sophisticated augmentation techniques, such as domain-specific GANs or time-series-based diffusion models, which may better capture the underlying structure of glucose signals. Additionally, exploring transfer learning from larger, related datasets may also provide a path to improve model performance without the immediate need for extensive new data collection.

It is important to note that the performance of our ML models is influenced by both the early-stage nature of the biosensor and the limited dataset size. The biosensor’s quality is reflected in its low detection limit (≈0.001 ppb), high specificity, and clear ohmic resistance shifts, indicating that the sensor itself provides consistent signal changes for different glucose concentrations. However, due to the relatively small number of samples and the inherent class imbalance, the machine learning models face challenges in achieving high F1-scores, especially for rare (high glucose) samples. Nonetheless, the improved performance observed with Random Forest and SMOTE underscores that addressing data imbalance and refining preprocessing steps can yield notable gains. In future work, as sensor fabrication scales up and more data become available, we anticipate that the performance of our ML pipeline will further improve, allowing us to better distinguish the sensor’s intrinsic performance from the effects of limited training data.

It is worth noting that this study establishes a foundational approach for glucose concentration prediction using biosensor data, offering valuable insights into both preprocessing and modeling. The results suggest that even with limited data, preprocessing and careful model selection can achieve meaningful performance. Moreover, as the biosensor technology advances, generating larger datasets with improved consistency across sensors may become feasible, allowing these models to achieve their full potential. This work highlights the adaptability of machine learning frameworks to biosensor data and sets the stage for future studies to build upon, with the potential for more robust and generalizable models as more data becomes available.

Overall, the results suggest that Random Forest with SMOTE is a promising approach for this application given the limited data, offering an interpretable and robust model for glucose concentration classification. However, deep learning models such as CNN and LSTM provide an alternative approach, achieving competitive results without the need for explicit feature engineering. Future work should investigate the scalability of these models to larger datasets, particularly using improved synthetic data generation techniques to handle class imbalance effectively. Additionally, as the models are deployed on real-world biosensor data, further emphasis on inter-sensor calibration and sensor-specific preprocessing will be crucial for achieving consistent, reliable predictions across different biosensor devices.

## Figures and Tables

**Table 1:** Comparison of current work with other MIP-based glucose sensors.

Sensor	Limit of Detection	Linear Range	Sample Source	Ref.
Presented Work	0.001 ppb	0.001 ppb–90 ppm	Aerosol	
MIPs on Al-PVC substrate	19.4 μM	0.0194 mM–0.3300 mM	PBS	[8]
Graphene Oxide-MIPs	0.02 μm	0.01 mM–6 mM	PBS	[9]
MIPs on Au QCM electrode	0.07 mM	0.07 mM–8 mM	Carbonate Buffer	[10]
Fe3O4@AuNC MIPs	5.0 μM	10.0 μM–5.0 mM	PBS	[11]
MIP-based SPCE	0.19 ± 0.15 μM	0.32 μM–1.0 mM	PBS	[12]

**Table 2:** Model performance evaluation based on Accuracy, F1-Score, Precision, and Recall.

Model	Preprocessing	Accuracy	F1-Score	Precision	Recall
CNN	None	0.6778	0.5138	0.5597	0.4904
CNN	Minority Oversampling	0.6653	0.5211	0.5299	0.5164
CNN	SMOTE	0.6653	0.4760	0.4857	0.4700
CNN	GAN	0.5858	0.4775	0.4852	0.4972
LSTM	None	0.6778	0.5116	0.5326	0.4982
LSTM	Minority Oversampling	0.6402	0.4874	0.4786	0.5074
LSTM	SMOTE	0.6025	0.4877	0.4820	0.5054
LSTM	GAN	0.6486	0.4973	0.4920	0.5183
Random Forest	Feature Extraction	0.7029	0.3695	0.3863	0.3740
Random Forest	Feature Extraction + Minority Oversampling	0.6820	0.4048	0.4471	0.3892
Random Forest	Feature Extraction + SMOTE	0.7197	0.4643	0.5090	0.5783
Random Forest	Feature Extraction + GAN	0.7029	0.3567	0.3795	0.3616
XGBoost	Feature Extraction	0.6778	0.2902	0.2925	0.3031
XGBoost	Feature Extraction + Minority Oversampling	0.6611	0.3899	0.4154	0.3817
XGBoost	Feature Extraction + SMOTE	0.6736	0.4083	0.4139	0.4178
XGBoost	Feature Extraction + GAN	0.6569	0.3831	0.3889	0.3881
SVM	Feature Extraction	0.7071	0.3719	0.3620	0.3879
SVM	Feature Extraction + Minority Oversampling	0.4979	0.4327	0.4398	0.4999
SVM	Feature Extraction + SMOTE	0.4477	0.3487	0.3603	0.3985
SVM	Feature Extraction + GAN	0.7029	0.2090	0.1795	0.2500

## Data Availability

The raw data supporting the conclusions of this article will be made available by the authors on request.
